# High frequency transcranial pulsed current stimulation improves quality of life in Parkinson’s disease: A randomized controlled trial

**DOI:** 10.21203/rs.3.rs-10834572/v1

**Published:** 2026-10-04

**Authors:** Zheng Li, Sandra Zhong, Lan Wang, Fengyi Hao, Wenjun Hong, Chunxia Zhao, Dongmei Ai, Zhigang Zhao, Ping Xiong, Tingting Sun, Kun Wang, Jessie Siew-Pin Leuk, Eng King Tan, Giuliana Rencricca, Esra Neufeld, Niels Kuster, Alvaro Pascual-Leone, Rong Xu

**Affiliations:** Nanjing Drum Tower Hospital; King’s College London; Nanjing Drum Tower Hospital; Chongqing Normal University; Nanjing Drum Tower Hospital; Nanjing Drum Tower Hospital; Nanjing Drum Tower Hospital; Nanjing Drum Tower Hospital; Nanjing Drum Tower Hospital; Nanjing Drum Tower Hospital; Nanjing Drum Tower Hospital; National Neuroscience Institute, Tan Tock Seng Hospital; National Neuroscience Institute, Singapore General Hospital; Swiss Federal Institute of Technology (ETH) Zurich; Foundation for Research on Information Technologies in Society (IT’IS); Swiss Federal Institute of Technology (ETH) Zurich; Hebrew SeniorLife; Nanjing Drum Tower Hospital

**Keywords:** Transcranial pulsed current stimulation, Parkinson’s disease, electric field modelling, Parkinson Disease Questionnaire-39, non-motor symptom, quality of life

## Abstract

Parkinson’s disease (PD) is a progressive multisystem disorder in which motor and non-motor symptoms impair quality-of-life. High-frequency transcranial pulsed current stimulation (tPCS) modulates network-level dysfunction and may serve as an adjunct therapy in PD. In a randomized controlled trial, 38 participants with Hoehn & Yahr stage I–III PD were randomized to active 400 Hz tPCS or sham for 4 weeks while receiving stable antiparkinsonian medication. Stimulation targeted bilateral dorsolateral prefrontal cortices and motor-cerebellar circuit. Primary outcome was the Parkinson’s Disease Questionnaire-39 Summary Index (PDQ-39 SI); secondary outcomes included motor and non-motor measures. Thirty participants completed the study with no serious adverse events. Compared with sham, active tPCS significantly improved PDQ-39 SI (p = 0.017; partial η^2^ = 0.19) and reduced non-motor symptom burden (NMSS, p = 0.005). High frequency tPCS was safe, well tolerated, and improved quality of life in PD, warranting investigation in larger trials.

**Trial registration:**

Chinese Clinical Trial Registry ChiCTR2000041517, Date of registration Dec 27, 2020 (URL https//www.chictr.org.cn/showprojEN.html?proj=66622)

## Introduction

Parkinson’s disease (PD) is the fastest-growing neurodegenerative disorder worldwide^1^. It is characterized by the progressive degeneration of dopaminergic neurons in the substantia nigra pars compacta and aggregation of misfolded α-synuclein, resulting in the cardinal motor features of bradykinesia, rigidity, tremor, and postural instability^2^. However, beyond motor dysfunction, PD is increasingly recognized as a multisystem disorder, with diverse non-motor symptoms often posing a greater burden than motor symptoms and substantially impairing quality of life^3^. As there is currently no curative treatment, treatment focuses on alleviating symptoms, minimizing disability, and optimizing patients’ quality of life^4^.

Dopamine replacement therapy remains the standard therapeutic approach for PD^5^. Dopaminergic medications can provide substantial relief of motor symptoms^4^, but their therapeutic benefit often declines with disease progression, and long-term use is frequently complicated by levodopa-induced complications, dyskinesias and motor fluctuations^6^. Furthermore, effects on non-motor symptoms are variable and often limited^7,8^, partly because optimal dopaminergic signaling differs across motor and non-motor circuits^9^. In frontostriatal networks, dopamine exhibits an inverted-U dose–response relationship, whereby both hypo- and hyperdopaminergic states are associated with compromised cognitive function^10^. Consequently, medication doses optimized for motor control may inadequately treat, or even exacerbate, non-motor symptoms in PD^11^.

The limitations of dopaminergic therapy have prompted increasing interest in adjunctive neuromodulation strategies that target dysfunctional neural circuits in PD. Deep brain stimulation is an established treatment for refractory symptoms^12^ but its invasive nature and surgical risks limit its applicability. Non-invasive brain stimulation has therefore emerged as a promising alternative. Repetitive transcranial magnetic stimulation (rTMS) has shown beneficial effects on motor manifestations and selected non-motor domains^13,14^, but responses are highly variable and its reliance on repeated clinic-based sessions limits accessibility. Transcranial electrical stimulation (tES) offers a more portable and user-friendly alternative with a favorable safety profile. Among tES modalities, transcranial direct current stimulation (tDCS) has been the most extensively investigated in PD with early trials reporting modest improvements in gait, balance, tremor, and bradykinesia^15,16^. However, evidence for non-motor benefits remains inconsistent. Several small studies suggested tDCS benefits non-motor symptoms^15,16^, but recent meta-analyses failed to demonstrate robust advantages over sham^17^. These findings highlight the need for more effective neuromodulation approaches.

High-frequency transcranial pulsed current stimulation (tPCS) is an emerging tES modality that delivers low-intensity pulsed currents at high frequencies (typically 100–500 Hz; ≤2 mA average current) to modulate neuronal activity in targeted brain regions^18–20^. Unlike tDCS, which delivers a continuous constant current, high-frequency tPCS delivers intermittent trains of brief, high-amplitude pulses separated by periods of no current. This pulsed waveform has been reported to produce stronger neurophysiological effects and recruit a broader range of sensory, motor, and nociceptive fibers at average currents as low as 0.3 mA^18^. The rapid onset and low duty cycle of each pulse also produces large transient changes in the extracellular electric field (dE/dt) and high peak exposure strengths, enhancing the polarization, synchronization, and entrainment impact on neuronal and network dynamics. Supporting this, computational modelling using detailed neuronal models demonstrated that high-frequency tPCS induced four-to-eight-fold greater somatic polarization of cortical pyramidal neurons and interneurons, and approximately three-fold greater modulation of spontaneous Purkinje-cell firing, than sinusoidal transcranial alternating current stimulation (tACS) at matched root mean square (RMS) current^21^.

Preclinical studies further suggest that high frequency pulsed electrical stimulation (100–500 Hz) can suppress neuronal firing through partial axonal conduction block^22–24^ and promote network desynchronization^25,26^. These properties may be particularly relevant to PD, where aberrant cortical–subcortical connectivity^27^ and excessive beta-band synchrony within the basal ganglia–thalamocortical circuits^28^ are implicated in both motor and non-motor manifestations. Prior high frequency tPCS studies have reported improvements in motor function in PD^29^ and non-motor benefits in children with autism spectrum disorder^20^.

We therefore hypothesized that adjunctive 400-Hz tPCS in patients with PD on optimized medication regimen, would improve motor and non-motor symptoms and, thus quality of life, presumably by modulation of dysfunctional neural networks. The 39-item Parkinson’s Disease Questionnaire Summary Index (PDQ-39 SI) was selected as the primary outcome because it provides a measure of health-related quality of life (HRQoL)^30^, capturing the disease-specific impact on physical, cognitive, and emotional well-being, daily functioning, and social participation, and thus more relevant for evaluating the therapeutic impact of 400Hz tPCS on patients’ quality of life. Secondary outcomes were various motor and non-motor scales to investigate the domain specific effects of adjunctive 400Hz-tPCS in PD.

## Results

Forty-three participants were screened: five did not meet inclusion / exclusion criteria due to presence of severe depression (*n* = 2) or change of anti-Parkinsonian medication within the last month (*n* = 3) and were excluded. Thirty-eight participants were randomly assigned either to active-tPCS (n = 19) or sham stimulation (n = 19). During the trial, one participant from the active-tPCS and four from the sham group withdrew before the first treatment due to unforeseen COVID-19 travel constraints, and another three from the sham group withdrew after the first session due to inconvenient schedules (n = 3). Thirty participants (19 males, 11 females; mean age 65 ± 8 years, range 51–76 years) completed the study, yielding a retention rate of 90.9% (30/33). Figure 1 summarizes participant flow.

### Assessment of electric-field exposure

Figure 2a and 2b show cumulative histograms of cerebral and cerebellar grey-matter electric-field (E-field) exposure at the peak of the tPCS pulse for both trial montages, together with brain-surface distributions of the normal E-field component and total E-field magnitude. For Montage 1 (anode: left dLPFC, cathode: right dLPFC), the 99th percentile E-field reached 6.8 V/m in the bilateral frontal cerebral grey-matter. For Montage 2 (anode: vertex; cathode: mid-cerebellar hemisphere), cerebral and cerebellar exposures reached 5.7 and 6.3 V/m respectively, with peak E-fields occurring directly beneath the electrodes.

### Safety of 400Hz tPCS

Across 603 tPCS sessions administered, no serious adverse events occurred. In the active-tPCS group, three participants reported transient scalp pain during the first session, and one reported a single episode of dizziness during Week 1. All events were mild, self-limited, and did not recur. This corresponded to approximately one adverse event per 150 treatment sessions. No adverse events occurred in the sham group, and the between-group difference was not statistically significant (Fisher’s exact test, *P* = 0.13; Supplement 3, eTables 2a–2b).

### Baseline demographics and clinical characteristic between groups

The demographic and clinical characteristics of the active-tPCS and sham participants are reported in Table 1. There were no statistically or clinically significant differences between groups at baseline.

### Effect of 400Hz tPCS on Primary and Secondary Efficacy Outcomes

Post-treatment between-group comparisons were performed using rank ANCOVA adjusted for corresponding baseline values given the small sample size and non-normal data distributions. The prespecified primary outcome, PDQ-39 SI, improved significantly in the active-tPCS group, with a median reduction of 14 points (baseline median: 20.5 [8.8,30.8]; post-treatment median: 6.5 [2.2,15.0]). In contrast, the sham group had a 2-point reduction in PDQ-39 SI (baseline median: 13.5 [10.2,23.8]; post-treatment median: 11.5 [8.0, 14.8]). Between-group rank ANCOVA revealed a significantly greater reduction in PDQ-39 SI following active tPCS (adjusted mean rank difference = − 5.8; 95% CI, − 10.5 to − 1.1; *P* = 0.017; partial η^2^ = 0.19). A sensitivity analysis was conducted to exclude a baseline outlier (> 1.5×IQR) in the active-tPCS. Results show that the active-tPCS group’s median PDQ-39 SI reduction became 9 points (baseline: 15.0 [8.0, 30.0] to posttreatment: 6.0 [2.0, 15.0]), while the between-group rank ANCOVA remained significant (adjusted mean rank difference = − 5.65; 95% CI, − 10.23 to − 1.07; *P* = 0.018; partial η^2^ = 0.20).

In the secondary outcomes, overall disease burden, assessed using the UPDRS total score, improved significantly with active-tPCS compared with sham (adjusted mean rank difference = − 4.3; 95% CI, − 7.5 to − 1.1; *P* = 0.011; partial η^2^ = 0.22). Among the UPDRS subscales, only the UPDRS I (Non-motor Experiences of Daily Living) score demonstrated a significant between-group difference, favoring active tPCS (adjusted mean rank difference = − 6.3; 95% CI, − 11.5 to − 1.1; *P* = 0.020; partial η^2^ = 0.18). No significant between-group differences were observed for the remaining UPDRS subscales, including UPDRS III motor scores. Following Bonferroni correction, the between-group difference in UPDRS I no longer reached statistical significance (corrected *P* = 0.081) whereas the difference in the UPDRS total score (corrected *P* = 0.042) remained significant.

Non-motor symptom burden measured by the NMSS also improved significantly following active tPCS. The active-tPCS group demonstrated a median reduction of 11.5 points in NMSS (baseline median: 24.0 [9.2, 38.0]; post-treatment median: 12.5 [2.5, 16.0]), whereas the sham group showed slight increase of 0.5 points (baseline median: 15.5 [10.2, 19.0]; post-treatment median: 16.0 [6.8, 18.2]). Rank ANCOVA adjusting for baseline NMSS scores confirmed a significant between-group difference in favor of active tPCS (adjusted mean rank difference = − 5.85; 95% CI, − 9.81 to − 1.89; *P* = 0.005; partial η^2^ = 0.25). As the active-tPCS group contained a baseline NMSS outlier (> 1.5×IQR), a sensitivity analysis was conducted excluding it. Results showed that the active-tPCS group’s median NMSS reduction became 10 points (baseline: 22.0 [9.0, 32.0] to post-treatment: 12.0 [2.0, 16.0]), while the between-group rank ANCOVA remained significant (adjusted mean rank difference = − 5.80; 95% CI, − 9.85 to − 1.76; *P* = 0.007; partial η^2^ = 0.25). No significant between-group differences were observed for other secondary outcomes. Post-treatment rank ANCOVA results are presented in Table 2.

Individual trajectory plots for PDQ-39 SI and NMSS from baseline to post-treatment, after excluding outliers, are shown in Fig. 3 and Fig. 4. They show the steeper decline in the group median for the active-tPCS compared to the sham group, with most individual participants in the active-tPCS group trending downward, while sham participants showing more variable, largely flat trajectories. These patterns are consistent with the significant between-group treatment effects observed for PDQ-39 SI (*P* = 0.018) and NMSS (*P* = 0.007) in the sensitivity analyses using rank ANCOVA.

### Exploratory correlations between changes in PDQ-39 SI and other outcomes

An exploratory Spearman correlation analysis was conducted between PDQ-39 SI and the various motor and non-motor outcome variables. Results showed that improvement in PDQ-39 SI scores in the active-tPCS group was significantly correlated with improvement in NMSS scores (ρ = 0.570, *P* = 0.014) only. No significant correlations were observed for the other outcome variables. Results of the exploratory correlation analysis are presented in Table 3.

## Discussion

The present study is a single-centre, double-blind, sham-controlled randomized pilot trial that evaluated the safety and efficacy of adjunct high-frequency (400-Hz) tPCS in individuals with mild to moderate PD receiving stable antiparkinsonian medications. Throughout the study, no serious adverse events occurred in either group. Mild adverse events were more frequent in the active tPCS group, including 3 episodes of transient scalp pain and 1 episode of mild dizziness, but overall adverse event incidence did not differ significantly between groups (4/18 vs 0/12; *P* = 0.130). The repeated tPCS sessions had a high adherence rate (90.9%). These findings support the safety and tolerability of 400Hz tPCS in individuals with PD.

Post-treatment, between-group rank ANCOVA adjusted for baseline scores demonstrated a significant improvement in PDQ-39 SI scores with active 400-Hz tPCS group compared with sham, with large effect size (adjusted mean rank difference = − 5.8; 95% CI, − 10.5 to − 1.1; *P* = 0.017; partial η^2^ = 0.19). Sensitivity analysis excluding an outlier in the active group revealed that the between-group difference was robust and remained significant (*P* = 0.018; partial η^2^ = 0.20), indicating that the effect was not driven by this observation.

The improvement in PDQ-39 SI was accompanied by significant between-group differences in overall disease severity as measured by the UPDRS total score (P = 0.011; partial η^2^ = 0.22) and non-motor symptom burden as measured by the NMSS score (P = 0.005; partial η^2^ = 0.25), with large effect sizes and favoring active tPCS. Among the UPDRS subdomains, only Part I (non-motor aspects) showed a significant between-group difference (*P* = 0.020), that, however, did not remain significant after correction for multiple comparisons. Nonetheless, this pattern of change is consistent with the NMSS and suggests that the observed HRQoL benefit following 400Hz-tPCS may have been more closely associated with reduction in non-motor symptoms. Supporting this, exploratory analysis within the active-tPCS group demonstrated that improvements in PDQ-39 SI were significantly correlated with reductions in NMSS scores (*P* = 0.014), but not with changes in other outcome measures. These findings are consistent with prior evidence associating changes in NMSS scores with clinically meaningful changes in the PDQ-39 SI^32^.

In contrast, the HRQoL benefit observed with 400-Hz tPCS in the present study differs from findings reported for other forms of transcranial electrical stimulation in PD. A cross-sectional study found no significant difference in PDQ-39 SI between patients previously exposed or unexposed to tDCS^33^, and a recent meta-analysis of six tDCS studies found no significant improvement in PDQ-39 SI despite benefits in specific activities of daily living^34^. Notably, in the present study, active tPCS produced a median 9-point reduction in PDQ-39 SI after exclusion of outliers, which is approximately 1.9 times the published MCID of 4.72 points^35^, whereas scores remained largely unchanged in the sham group. Thus, the observed improvement in HRQoL was not only statistically significant but also clinically meaningful.

Given that 90% of participants in the present study had relatively preserved baseline HRQoL, with PDQ-39 SI scores comparable to those reported in early-stage PD^36^, the observed improvement in PDQ-39 SI following 4 weeks of active tPCS may have been mediated by improvements in non-motor symptoms that strongly influence patient-perceived well-being, including mood disturbances such as anxiety and depression, and cognitive difficulties such as impaired concentration and memory complaints, which have been found to be important determinants of HRQoL in early PD^3^. However, the small sample size precluded secondary analyses of individual NMSS domains and their associations with PDQ-39 SI. Larger studies are therefore needed to determine which specific non-motor domains are most strongly associated with improvements in HRQoL following 400Hz tPCS.

Although the mechanisms by which high frequency tPCS improves HRQoL in PD remain incompletely understood, one potential explanation may lie in the magnitude and spatial targeting characteristics of the 400-Hz tPCS-induced intracranial electric field. Compared to previous tDCS studies, which generally reported induced intracranial electric fields below 1 V/m^37–39^ at an average current magnitude of 1 mA, our modelling of Montage 1 predicted 99th-percentile E-field magnitude exceeding 6 V/m in bilateral frontal grey matter at the same average total current intensity. Given evidence that predictions from personalized electric-field dosimetry correlate with the inter-individual variability in tES responses and influences neuroplastic effects^38,39^, it is possible that the greater field magnitude generated by 400-Hz tPCS more robustly engaged the bilateral DLPFC and facilitated the modulation of prefrontal networks implicated in non-motor symptoms. Additionally, with the anode positioned over the left DLPFC and the cathode over the right DLPFC, 400-Hz tPCS may have promoted leftlateralized prefrontal activity involved in cognitive and executive control^40–42^ while reducing abnormal right prefrontal activity associated with dysfunctional emotional processing, including attentional difficulties and excessive rumination^43–45^. Furthermore, the temporal characteristics of 400-Hz tPCS are mechanistically distinct from those of conventional direct current stimulation. High-frequency pulse trains have been shown to promote neural desynchronization^25,26^, raising the possibility that 400-Hz tPCS disrupted pathological hyper-synchronization in PD^28^ including network abnormalities implicated in non-motor manifestations^46^, thereby contributing to improvements in HRQoL.

Despite Montage 2 generating peak electric-field magnitudes comparable to those of Montage 1, we observed no significant between-group differences in motor-specific outcomes, including UPDRS Part III, Timed Up and Go, and Berg Balance Scale scores. One possible explanation is that the greater inter-electrode distance between the vertex anode and midline cerebellar cathode produced a more spatially distributed electric field, potentially limiting effective modulation of the cerebello–basal ganglia–cortical motor pathway^47^ implicated in PD motor symptoms. Alternatively, any motor effects of 400-Hz tPCS may have been less readily detectable against the established therapeutic effects of concomitant dopaminergic medications which were maintained throughout the trial, particularly as assessments were not performed during OFF periods and the cohort predominantly had mild PD with limited motor impairment.

This study has several limitations. First, the final group sizes were imbalanced because of differential attrition between the sham and active-tPCS groups. Four participants assigned to sham and one assigned to active tPCS withdrew before receiving the first intervention due to COVID-19-related travel restrictions that impeded attendance to the study site. An additional three participants in the sham group withdrew after the first treatment session without completing post-treatment assessments, limiting the feasibility of an intention-to-treat analysis and resulting in unequal group sizes in the final analysis. To mitigate potential bias arising from baseline imbalance in this relatively small sample, between-group comparisons were performed using rank ANCOVA with adjustment for the corresponding baseline outcome values, an approach selected in view of the non-normal distribution of several outcomes.

A further limitation is that participant blinding assessment was not formally evaluated using a blinding questionnaire. However, the risk of unblinding was low because adverse events were mild, expected and comparable between groups, and participants were prevented from interacting during study procedures. In addition, all study personnel interacting with participants remained blinded to treatment allocation.

A third limitation is that although Montage 1 was designed primarily to engage prefrontal networks implicated in non-motor symptoms and Montage 2 to engage cerebello–basal ganglia–cortical motor pathways, the two montages were administered consecutively within each treatment session. Their individual contributions to the observed clinical effects therefore cannot be disentangled and remains uncertain.

In conclusion, this pilot randomized controlled trial provides preliminary evidence that adjunctive 400-Hz tPCS is safe, well tolerated, and may produce clinically meaningful improvements in HRQoL in individuals with PD. The improvement in HRQoL was accompanied by reductions in NMSS and UPDRS total scores, suggesting that the HRQoL benefit may have been mediated through improvement in non-motor symptoms. This finding is clinically relevant because non-motor symptoms often remain insufficiently addressed by standard antiparkinsonian therapies, which are primarily optimized for motor symptom control. Collectively, these results support further investigation of high frequency tPCS as an adjunctive therapeutic approach for PD, particularly for alleviating non-motor symptoms which are closely associated with quality of life in PD. Larger, adequately powered multicenter RCTs are needed and warranted to confirm the effects of 400-Hz tPCS on HRQoL in PD, identify the non-motor domains underlying these effects, and optimize electric-field targeting of motor networks.

## Methods

### Study Design

A four-week, single-center, double-blind, randomized, sham-controlled clinical trial was conducted at Nanjing Drum Tower Hospital, Jiangsu Province, China. Eligible participants who met criteria and provided written informed consent were randomized 1:1 to receive either active 400 Hz tPCS or sham stimulation. The study was approved by the Ethics Committee of Nanjing Drum Tower Hospital (approval No. 202230702), registered with the Chinese Clinical Trial Registry (ChiCTR2000041517), and conducted in accordance with the Declaration of Helsinki.

### Participants

Participants with PD were recruited between January 31, 2021 and March 15, 2023 from the neurology and rehabilitation outpatient clinics of Nanjing Drum Tower Hospital. PD was diagnosed according to the 2015 MDS criteria and Chinese guidelines^2,48^, requiring bradykinesia plus at least one of resting tremor, rigidity, or postural instability, a clear levodopa response, and absence of clinical red flags for atypical parkinsonism (e.g., rapid progression, early severe autonomic failure, early dementia).

Inclusion criteria were age 50–85 years; male or female, Hoehn and Yahr stage 1–3; Mini-Mental State Examination (MMSE) score > 24; stable antiparkinsonian medication for ≥ 1 month; ability to complete study procedures; and provision of written informed consent. Exclusion criteria included major psychiatric or intellectual disability; unstable cardiovascular, pulmonary, renal, or cerebrovascular disease; other neurological or musculoskeletal causes of motor impairment; history of seizures or recent craniotomy; implanted electronic or metallic devices; pregnancy; prior exposure to non-invasive brain stimulation within 3 months; concurrent participation in another clinical trial; or any condition expected to require surgery or intensive medical treatment during the study.

### Sample size calculation

The original sample size was 72 participants (36 per group); however, COVID-19 restrictions in Nanjing substantially impeded recruitment within the approved study period. Consequently, the study was completed as a pilot randomized controlled trial to inform a future powered RCT, based on previous PDQ-39 data in PD^49^, with a target of 36 evaluable participants (18 per group).

### Randomization

A total of 38 eligible participants were randomized to active or sham stimulation using a computer-generated sequence prepared by an independent statistician not involved in recruitment, intervention delivery, outcome assessment, or data analysis. Allocation concealment was maintained using sequentially numbered, opaque, sealed envelopes opened only after baseline assessments. The randomization list was securely maintained by an independent administrator.

### Blinding

Participants, device operators, and outcome assessors were blinded to treatment allocation. Active and sham devices were identical in appearance and activated using preconfigured group-specific codes, which were entered by a study member not involved in treatment delivery, outcome assessment, or data analysis, thereby maintaining operator blinding. To minimize interaction between participants, visits were scheduled at least two hours apart, and participants were instructed not to discuss their treatment experiences. All 20 sessions were conducted in the same treatment environment. All participants were tPCS-naïve and were informed that transient stimulation-related sensations, including mild scalp pain, headache, skin redness, and itching, could occur with either active or sham stimulation to minimize potential unblinding.

### Intervention

Stimulation was delivered with a transcranial pulsed current stimulator (YQ-D1222, Yiqi Biotechnology Ltd., China). Participants attended 20 daily sessions of 20 minutes each over four consecutive weeks (Monday–Friday). For each participant, all sessions were applied at a consistent time. 400Hz tPCS was delivered using two circular silver–fiber cotton electrodes (12.56 cm^2^) soaked in 0.9% saline and secured to the scalp with a head strap. Two montages were applied sequentially in each session, each for 10 min, separated by a 5-min interval (total stimulation time 20 min/day). Montage 1 positioned the anode over the left dorsolateral prefrontal cortex (L-DLPFC, EEG:F3) and the cathode over the right dorsolateral prefrontal cortex (R-DLPFC; EEG:F4) to target cognitive–affective non-motor circuits^50,51^; Montage 2 placed the anode over the vertex (EEG:Cz) and the cathode over the mid-cerebellar region (EEG:Iz) to engage cerebello–basal ganglia–cortical motor pathways^47^. Active tPCS comprised a 10-s ramp-up to 1 mA (time-averaged current), maintained for each 10-min block, followed by a 5-s ramp-down. Sham stimulation comprised an identical 10-s ramp-up followed immediately by a 5-s ramp-down to zero, with no subsequent stimulation. Participants were permitted to continue their usual anti-parkinsonian medications, including levodopa preparations (e.g., Madopar), dopamine agonists (e.g., pramipexole), anticholinergics (e.g., trihexyphenidyl), and MAO-B inhibitors (e.g., rasagiline). Changes in medications during the study were not permitted. All stimulation sessions were administered by rehabilitation doctors and physical therapists, who remained blinded to group allocation throughout the study.

### Electric field Computational Exposure Assessment

To quantify tPCS-induced electric fields in the cerebellum and cerebral hemispheres, electromagnetic simulations were performed using Sim4Life V9.6 (ZMT, Zurich, Switzerland) with the high-resolution MIDA anatomical model^52^ that distinguishes153 tissue structures. Cylindrical 4-cm-diameter electrodes were positioned in locations corresponding to the clinical montages. Simulations used the Sim4Life’s ohmic-current-dominated electro-quasistatic solver to obtain the E-field, with rectilinear discretization employing a maximum grid spacing of 0.5 mm (~ 76 million elements), and tissue conductivities were assigned according to the IT’IS Tissue Properties Database v4.2.^53^. Electrodes were assigned Dirichlet boundary conditions (− 1 and + 1 V), with zero-flux Neumann conditions at the anatomical model surface, and the resulting fields were normalized to the peak total current (20-mA during pulse). Solver convergence was ensured by requiring a residual reduction of 12 orders of magnitude. See Fig. 5.

### Outcome Measures

The primary objective was to evaluate the effect of 400 Hz tPCS on QoL, assessed with the Chinese version of the Parkinson’s Disease Questionnaire-39 (PDQ-39)^54^, a validated disease-specific self-report measure. The PDQ-39 comprises 39 items across eight domains (Mobility, Activities of Daily Living, Emotional Well-being, Stigma, Social Support, Cognition, Communication, Bodily Discomfort), each rated on a 5-point Likert scale (0 = never to 4 = always), with higher scores indicating poorer HRQoL. Domain scores and the PDQ-39 Summary Index (PDQ-39 SI) were calculated according to standard procedures and transformed to a 0–100 scale, with the PDQ-39 SI serving as the primary outcome.

Secondary outcomes examined the effects of 400 Hz tPCS on motor and non-motor symptoms. Parkinsonian severity was evaluated with the Chinese version of the Movement Disorder Society–Unified Parkinson’s Disease Rating Scale (MDS-UPDRS)^55^, comprising four parts (I–IV), with higher scores indicating greater impairment. Part I (non-motor experiences of daily living) and Part III (motor examination) were used for non-motor and motor symptom subgroup analyses, respectively. Non-motor symptom burden was further assessed with the Chinese version of the Non-Motor Symptoms Scale (NMSS)^56^, which rates symptoms across nine domains; frequency and severity scores were combined to yield a total score (0–360), with higher scores reflecting greater non-motor burden. Depressive symptoms were measured with the 17-item Chinese Hamilton Depression Rating Scale (HAMD-17)^57^ with higher total scores indicating more severe depression. Fatigue was assessed using the Chinese Parkinson Fatigue Scale-16 (PFS-16)^58^, and the mean item score was used as the summary measure. Balance was evaluated with the Chinese Berg Balance Scale (BBS)^59^, and functional mobility with the Timed Up and Go Test (TUGT)^60^; longer TUGT times indicated poorer mobility.

Baseline assessments were performed at Week 0. Post-treatment outcomes were performed after the final stimulation session by trained neurologists and rehabilitation specialists blinded to treatment allocation.

### Safety monitoring

Participants were assessed during each stimulation session for discomfort, skin irritation at electrode sites, dizziness, headache, mood changes, or other adverse effects. Vital signs and general clinical status were checked before and after sessions, and participants were instructed to report any delayed or intersession adverse events. All safety events were systematically documented and reviewed by the study team to determine severity, seriousness, and potential relatedness to 400Hz tPCS or sham.

### Statistical Analysis

Statistical analyses were performed using IBM SPSS Statistics version 23.0 (IBM Corp., Armonk, NY, USA) and R version 4.2.2 (R Foundation for Statistical Computing, Vienna, Austria). Primary analyses were conducted in the per-protocol population, defined as participants who completed the intervention and provided post-treatment data. Baseline between-group balance was assessed comparing medians and IQRs using Wilcoxon rank-sum tests and by Fisher’s exact test for categorical variables. Normality of outcomes at baseline (T0) and post-treatment (T1) was assessed using the Shapiro–Wilk test (See Supplement 3, eTable 1).

Given the non-normal distribution of several outcomes and the need to adjust for baseline differences, post-treatment between-group comparisons were performed using rank analysis of covariance (rank ANCOVA) according to the Conover–Iman method^31^. For each outcome, T1 values were ranked across participants and analyzed using ANCOVA, with treatment group (active tPCS vs sham) as the between-subjects factor and the corresponding untransformed T0 value as a covariate. This rank-based approach provides a distribution-robust comparison while retaining adjustment for baseline outcome values. Adjusted mean rank differences with 95% confidence intervals and partial η^2^ effect sizes were reported.

PDQ-39 SI was prespecified as the primary outcome and was therefore tested without adjustment for multiplicity. Bonferroni correction was applied to the MDS-UPDRS total and component-score comparisons. For PDQ-39 SI and NMSS, sensitivity analyses were performed by repeating the rank ANCOVA after excluding baseline outliers, defined as values > 1.5 × IQR beyond the quartiles, to assess the robustness of the treatment effects. Exploratory associations between changes in PDQ-39 SI and other clinical outcomes within the active-tPCS group were evaluated using Spearman’s rank correlation coefficients. Between-group differences in adverse-event incidence were assessed using Fisher’s exact test. All tests were two-sided, with P < 0.05 considered statistically significant unless otherwise specified following multiplicity correction.

## Supplementary Material

This is a list of supplementary files associated with this preprint. Click to download.

- Supplement1TrialProtocolCombinedENCN.pdf
- Supplement2CONSORT2025checklistHighfrequencytranscranialpulsedcurrentstimulationimprovesqualityoflifeinParkinsonsdisease.pdf
- Supplement3eTablesHighfrequencytranscranialpulsedcurrentstimulationimprovesqualityoflifeinParkinsonsdisease.pdf

## Figures and Tables

**Figure 1:**
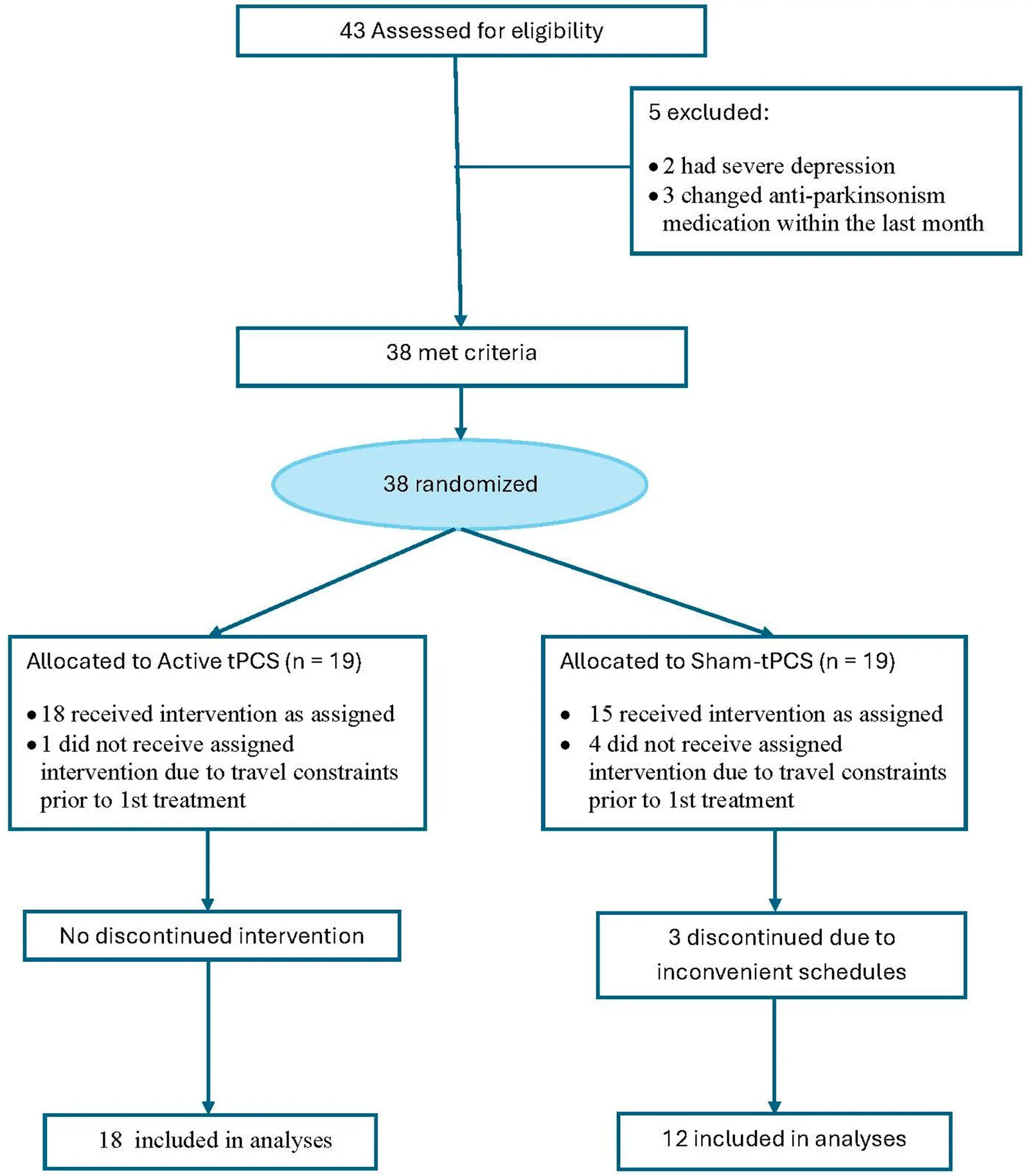
CONSORT flow diagram

**Figure 2:**
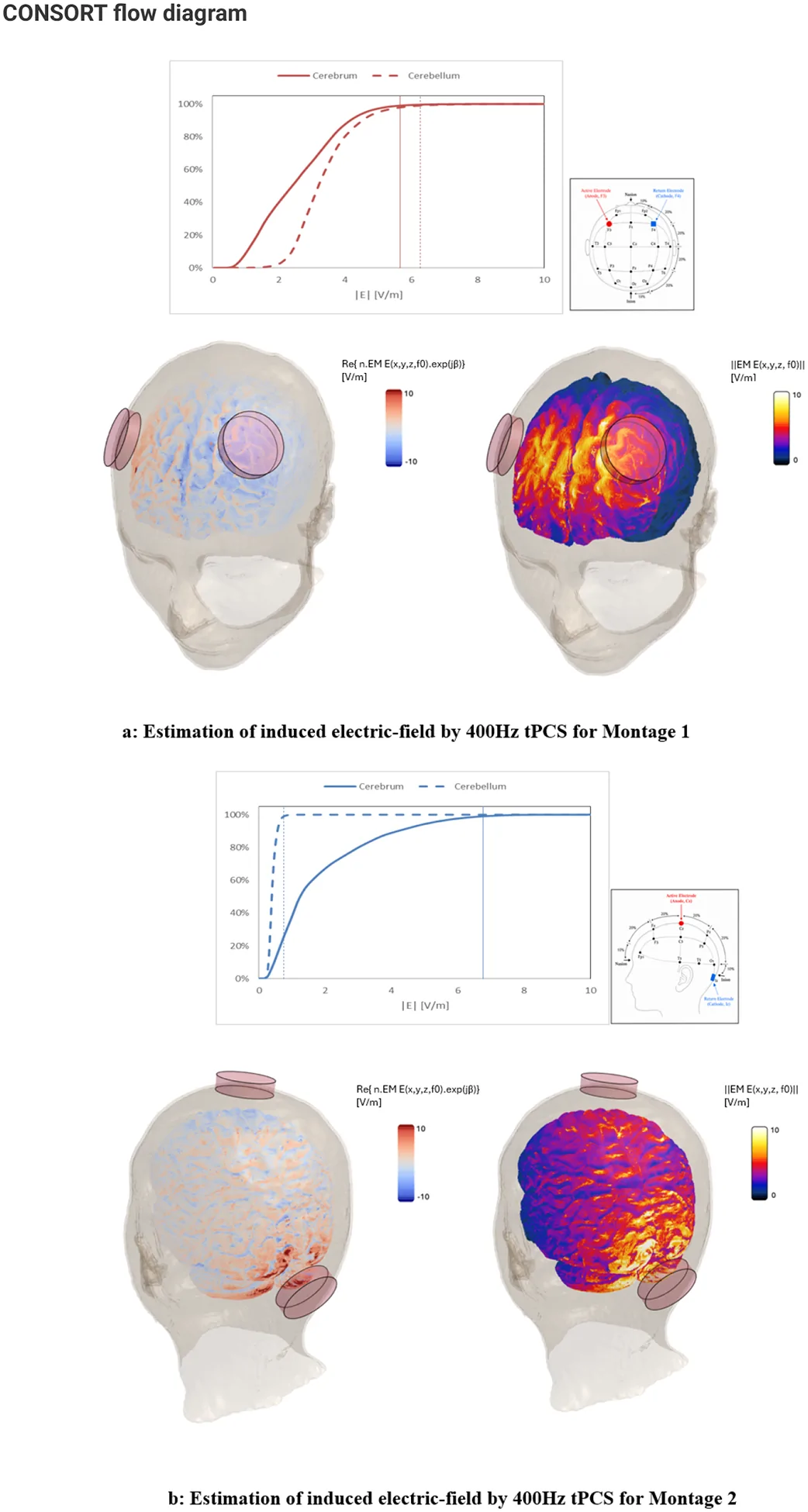
a: Estimation of induced electric-field by 400Hz tPCS for Montage 1 Cumulative E-field exposure histograms for cerebral grey matter, with vertical lines indicating the 99th percentiles for Montage 1. Corresponding brain-surface distributions of the normal E-field component (bottom left) and total E-field magnitude (bottom right). Figure 2b: Estimation of induced electric-field by 400Hz tPCS for Montage 2 Cumulative E-field exposure histograms for cerebral and cerebellar grey matter, with vertical lines indicating the 99th percentiles for Montage 2. Corresponding brain-surface distributions of the normal E-field component (bottom left) and total E-field magnitude (bottom right).

**Figure 3:**
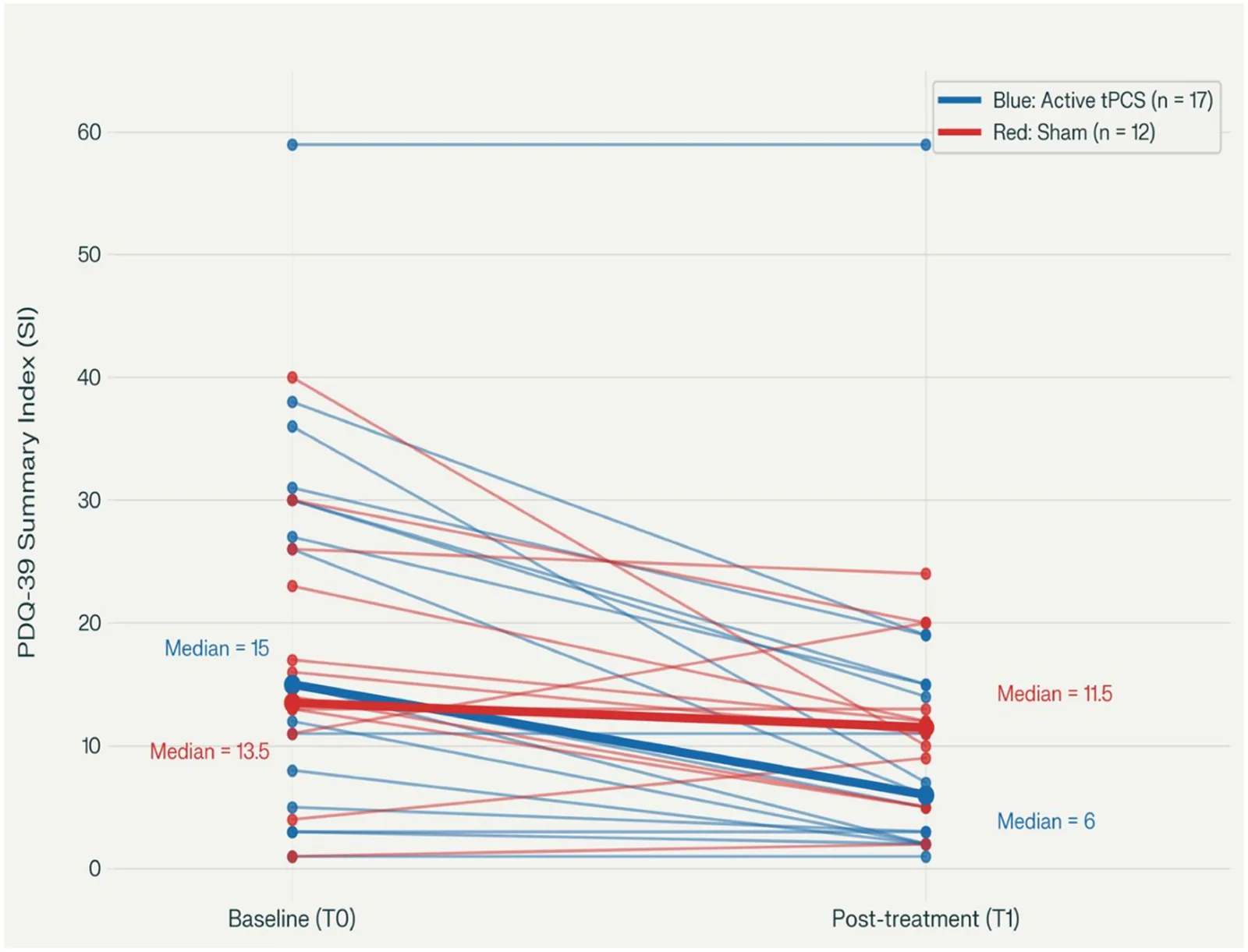
Individual subject trajectories in PDQ-39 SI scores from baseline to post-treatment (Sensitivity Analysis) Individual PDQ-39 SI trajectories for all 29 subjects (outlier NGH0009 in active-tPCS group excluded), with bold median lines overlaid. The active group’s median drops from baseline 15 to pos-treatment 6 (D60.0%), while the sham group’s median declines more modestly, from baseline 13.5 to post-treatment 11.5 (D14.8%). Lower PDQ-39 SI scores indicate symptom improvement.

**Figure 4:**
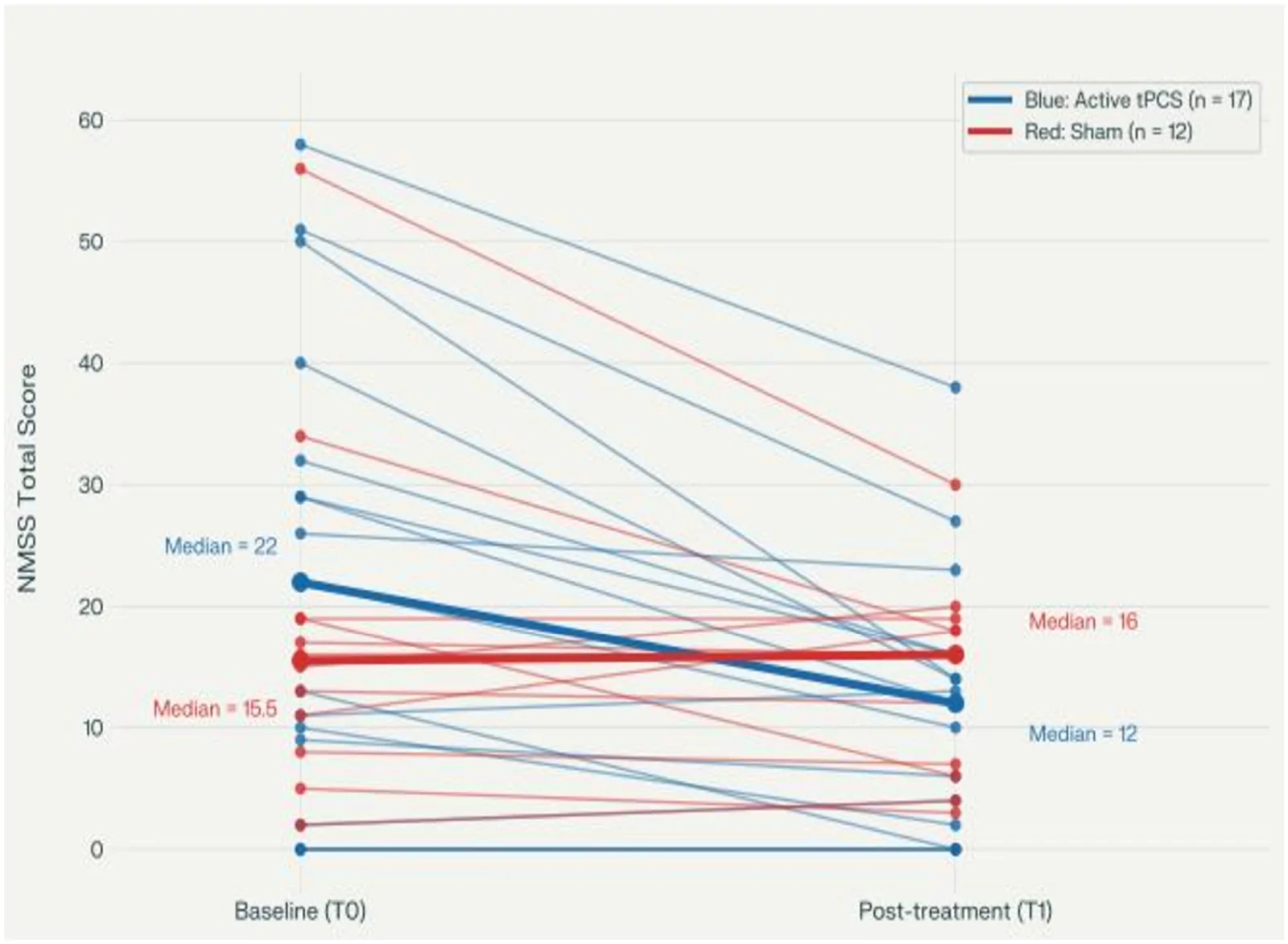
Individual trajectories in NMSS Total Score from baseline to post-treatment (Sensitivity Analysis) Individual NMSS trajectories for all 29 subjects (outlier NGH0027 in active-tPCS group excluded), with bold median lines overlaid. The active group’s median drops from baseline 22 to pos-treatment 12 (D45.5%), while the sham group’s median modestly worsen, from baseline 15.5 to post-treatment 16.0 (D 3.23%). Lower NMSS scores indicate symptom improvement.

**Figure 5:**
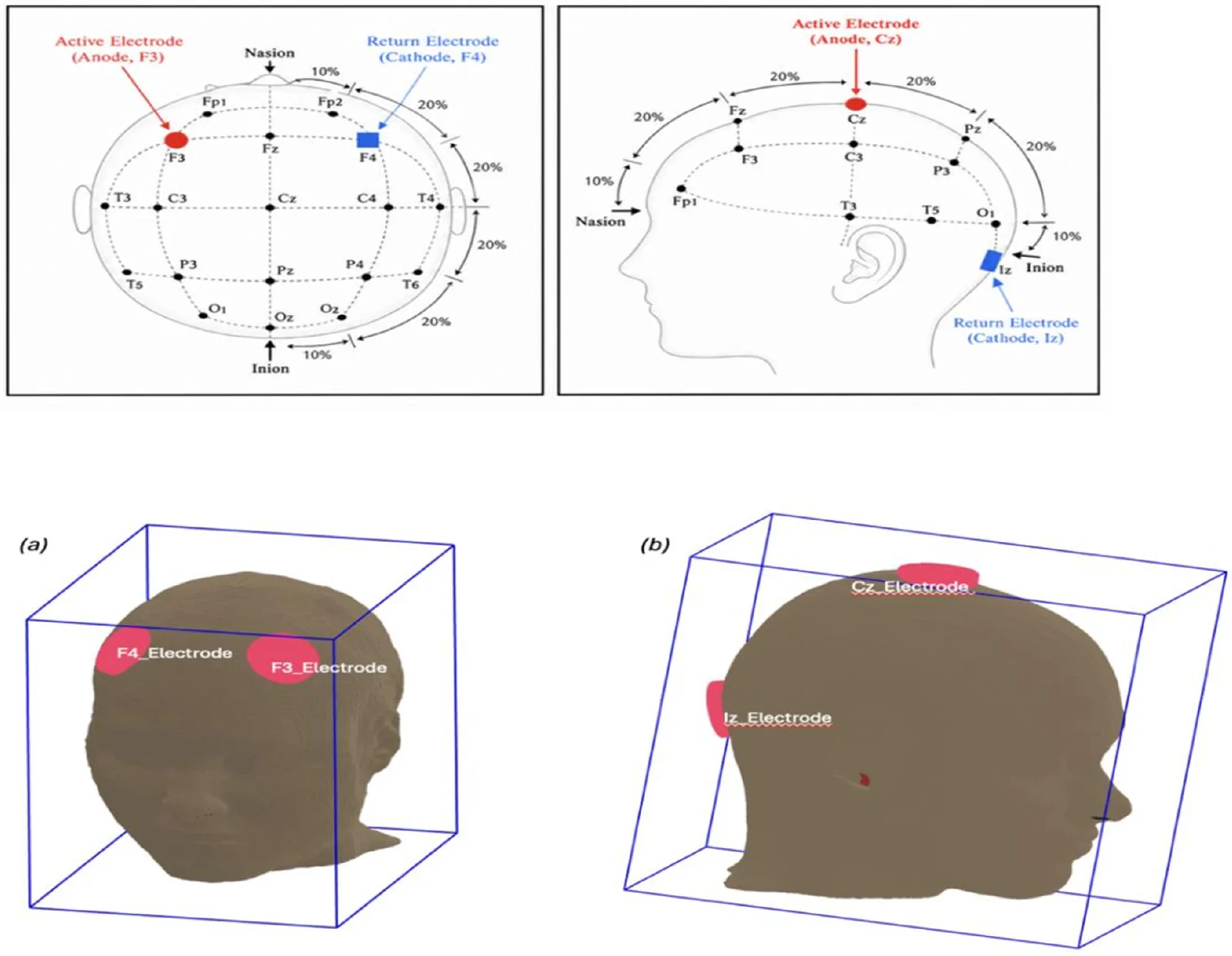
Electrode montage configurations for transcranial pulsed current stimulation (tPCS), shown on the 10–20 EEG system (top) and corresponding 3D voxel head models (bottom). Top row: Schematic placements on the 10–20 EEG system. *(Left)* Montage 1: the active electrode (anode, red circle) was placed over the left dorsolateral prefrontal cortex (L-dLPFC; site F3), and the return electrode (cathode, blue square) over the right dorsolateral prefrontal cortex (R-dLPFC; site F4). *(Right)*Montage 2: the active electrode (anode, red circle) was placed over the vertex (site Cz), and the return electrode (cathode, blue square) over the mid-cerebellar/inion region (site Iz). Bottom row: Corresponding 3D reconstructions on individual head models. (a) Montage 1, with electrodes positioned at F3 and F4. (b)Montage 2, with electrodes positioned at Cz and Iz.

**Table 1:** Baseline Demographics and Clinical Characteristics

Characteristic	Group			
	Overall (N = 30)	Active-tPCS (N = 18)	Sham-tPCS (N = 12)	P (Mann–Whitney U)
**Age**, median (IQR)	66.0 (57.2, 68.8)	67.0 (59.5, 68.8)	64.0 (57.0, 68.2)	0.396
**Sex, n (%)**				
Female	11/30 (36.7%)	8/18 (44.4%)	3/12 (25.0%)	0.442^1^
**Education years**, median (IQR)	9.0 (9.0, 12.0)	9.0 (9.0, 12.0)	12.0 (9.0, 15.0)	0.168
**MMSE**, median (IQR)	29.0 (27.0, 29.8)	29.0 (26.2, 29.8)	29.0 (28.0, 29.2)	0.680
**Modified H&Y stage**, median (IQR)	2.0 (1.5, 2.5)	1.8 (1.5, 2.4)	2.2 (1.9, 2.5)	0.187
**PDQ-39 SI**, median (IQR)	15.0 (8.8, 30.0)	20.5 (8.8, 30.8)	13.5 (10.2, 23.8)	0.458
**UPDRS Total**, median (IQR)	31.0 (19.2, 43.0)	33.0 (20.0, 58.5)	27.0 (21.2, 33.2)	0.219
**UPDRS I (Nonmotor)** median (IQR)	6.5 (3.0, 11.0)	9.0 (3.0, 13.5)	4.0 (3.0, 6.2)	0.115
**UPDRS II (ADL)**, median (IQR)	8.0 (6.0, 11.8)	8.0 (7.0, 12.5)	8.0 (3.8, 10.5)	0.457
**UPDRS III (Motor)**, median (IQR)	15.0 (9.2, 21.0)	17.5 (9.2, 29.2)	14.5 (10.5, 17.2)	0.525
**NMSS**, median (IQR)	16.5 (9.2, 31.2)	24.0 (9.2, 38.0)	15.5 (10.2, 19.0)	0.582
**HAMD**, median (IQR)	5.0 (2.2, 8.5)	5.5 (3.0, 9.8)	5.0 (1.8, 7.0)	0.241
**PFS**, median (IQR)	33.5 (28.5, 47.5)	32.5 (28.5, 52.5)	35.0 (30.5, 40.5)	0.983
**TUGT**, median (IQR)	10.4 (9.5, 13.1)	10.7 (9.7, 14.2)	10.2 (9.2, 12.1)	0.485
**BBS**, median (IQR)	55.0 (52.2, 56.0)	55.0 (51.5, 56.0)	55.0 (53.5, 56.0)	0.828

**Abbreviations**: SD, standard deviation; IQR, interquartile range; MMSE, Mini-Mental State Examination; H&Y, Hoehn and Yahr stage; PDQ-39, 39-item Parkinson’s Disease Questionnaire; UPDRS, MDS-Unified Parkinson’s Disease Rating Scale; NMSS, Non-Motor Symptoms Scale; HAMD, Hamilton Depression Rating Scale; PFS, Parkinson’s Fatigue Scale; TUGT, Timed Up and Go Test; BBS, Berg Balance Scale.

Given the non-normal distributions and small sample, P-values were derived from comparing medians and IQRs using Wilcoxon rank-sum test.

1 Fisher’s exact test (categorical variable).

**Table 2:** Post-treatment outcomes analyzed using Rank ANCOVA

Outcome measure	Median (IQR)		Adjusted Mean Rank^1^ (Mean ± SE)		Adjusted Mean Rank Diff Btw Grp (95% CI)	*F Statistic*	*P* value^1^	Effect Size
	Sham	Active-tPCS	Sham	Active-tPCS				
**PDQ-39 SI**	11.50 (8.0–14.8)	6.5 (2.2–15.0)	19.0 ± 1.8	13.2 ±1.4	−5.8 (−10.5,−1.1)	6.51	0.017*	0.19
**UPDRS TOTAL**	19.5 (15.0–26.0)	20.0 (11.5–34.2)	18.1 ± 1.2	13.8 ±1.0	−4.3 (−7.5, −1.1)	7.54	0.011*	0.22
**UPDRSI (Nonmotor)**	4.0 (3.8–6.0)	3.0 (1.2–6.8)	19.3 ± 1.9	13.0 ±1.5	−6.3 (−11.5, −1.0)	6.08	0.020*	0.18
**UPDRS II (ADL)**	8.0 (4.8–11.8)	6.0 (3.2–11.0)	17.6 ± 1.8	14.1 ±1.4	−3.5 (−8.2, 1.2)	2.32	0.140	0.08
**UPDRS III (Motor)**	9.5 (4.8–11.8)	7.5 (4.0–14.8)	16.7 ± 1.6	14.7 ±1.3	−2.0 (−6.3, 2.3)	0.88	0.356	0.03
**NMSS**	16.0 (6.8–18.2)	12.5 (2.5–16.0)	19.0 ± 1.5	13.2 ±1.2	−5.9 (−9.8, −1.9)	9.18	0.005*	0.25
**HAMD**	3.5 (2.0–5.2)	4.5 (2.0–6.8)	15.1 ± 2.2	15.8 ±1.8	0.7 (−5.1, 6.6)	0.07	0.798	0.00
**PFS**	39.0 (27.8–49.5)	33.5 (30.2–37.0)	17.3 ± 2.1	14.3 ±1.7	−3.0 (−8.5, 2.4)	1.32	0.261	0.05
**TUGT**	9.1 (8.6–10.5)	9.7 (8.6–13.8)	15.5 ± 1.7	15.5 ±1.4	0.1 (−4.5, 4.6)	0.00	0.978	0.00
**BBS**	55.0 (54.0–56.0)	55.5 (52.5–56.0)	14.5 ± 1.9	16.2 ±1.6	1.6 (−3.5, 6.7)	0.43	0.518	0.02

**Abbreviations**:

SE= Standard Error; CI= Confidence Interval; PDQ-39 SI = 39-item Parkinson’s Disease Questionnaire Summation Index; UPDRS = MDS-Unified Parkinson’s Disease Rating Scale; UPDRS I (Nonmotor) = MDS-Unified Parkinson’s Disease Rating Scale I; UPDRS II (ADL) = MDS-Unified Parkinson’s Disease Rating Scale II; UPDRS III (Motor) = MDS-Unified Parkinson’s Disease Rating Scale III; NMSS = Non-Motor Symptoms Scale; HAMD = Hamilton Depression Rating Scale; PFS = Parkinson’s Fatigue Scale; TUGT =Timed Up and Go Test; BBS =Berg Balance Scale

P-values were obtained using:

1 Rank ANCOVA, based on the method of Conover and Iman^31^. Bonferroni correction was applied across the MDS-UPDRS total score and its three subdomains, after correction, UPDRS I (Nonmotor) (p = 0.020) was no longer significant (See Supplement 3, eTable 3).

Partial n^2^ effect size : Small effect = ~ 0.01; Medium effect = ~ 0.06; Large effect = ≥ 0.14

**Table 3:** Exploratory Spearman Correlation analysis between Change in PDQ-39 SI and various outcomes in the Active-tPCS Group (N = 18)

(T1-T0)		Δ in PDQ SI
Δ in UPDRS Total	Spearman’s ρ	0.306
	P-value	0.217
Δ in UPDRS I (Nonmotor)	Spearman’s ρ	0.177
	P-value	0.483
Δ in UPDRS II (ADL)	Spearman’s ρ	0.096
	P-value	0.706
Δ in UPDRS III (Motor)	Spearman’s ρ	0.278
	P-value	0.264
Δ in NMSS	Spearman’s ρ	0.570
	P-value	0.014*
Δ in HAMD	Spearman’s ρ	0.358
	P-value	0.145
Δ in PFS	Spearman’s ρ	0.213
	P-value	0.396
Δ in TUGT	Spearman’s ρ	0.423
	P-value	0.081

## Data Availability

All datasets supporting the conclusions of this article are included within the article and its additional files.
